# Mutation Rates, Spectra, and Genome-Wide Distribution of Spontaneous Mutations in Mismatch Repair Deficient Yeast

**DOI:** 10.1534/g3.113.006429

**Published:** 2013-09-01

**Authors:** Gregory I. Lang, Lance Parsons, Alison E. Gammie

**Affiliations:** * Lewis-Sigler Institute for Integrative Genomics, Princeton University, Princeton, New Jersey 08544-1014; † Department of Molecular Biology, Princeton University, Princeton, New Jersey 08544-1014

**Keywords:** mismatch repair, mutation accumulation, mutation rate, homopolymeric runs, microsatellites

## Abstract

DNA mismatch repair is a highly conserved DNA repair pathway. In humans, germline mutations in *hMSH2* or *hMLH1*, key components of mismatch repair, have been associated with Lynch syndrome, a leading cause of inherited cancer mortality. Current estimates of the mutation rate and the mutational spectra in mismatch repair defective cells are primarily limited to a small number of individual reporter loci. Here we use the yeast *Saccharomyces cerevisiae* to generate a genome-wide view of the rates, spectra, and distribution of mutation in the absence of mismatch repair. We performed mutation accumulation assays and next generation sequencing on 19 strains, including 16 *msh2* missense variants implicated in Lynch cancer syndrome. The mutation rate for DNA mismatch repair null strains was approximately 1 mutation per genome per generation, 225-fold greater than the wild-type rate. The mutations were distributed randomly throughout the genome, independent of replication timing. The mutation spectra included insertions/deletions at homopolymeric runs (87.7%) and at larger microsatellites (5.9%), as well as transitions (4.5%) and transversions (1.9%). Additionally, repeat regions with proximal repeats are more likely to be mutated. A bias toward deletions at homopolymers and insertions at (AT)_n_ microsatellites suggests a different mechanism for mismatch generation at these sites. Interestingly, 5% of the single base pair substitutions might represent double-slippage events that occurred at the junction of immediately adjacent repeats, resulting in a shift in the repeat boundary. These data suggest a closer scrutiny of tumor suppressors with homopolymeric runs with proximal repeats as the potential drivers of oncogenesis in mismatch repair defective cells.

Mutations in DNA have far ranging consequences, from driving evolution to causing disease. DNA mismatch repair is a highly conserved process that maintains the fidelity of genomes by decreasing the mutation rate 100- to 1000-fold (Kunkel and Erie 2005). Mismatch repair proteins detect helical distortions or mismatches derived from exposure to mutagens (Stojic *et al.* 2004) during inexact replication of the genome (Hsieh and Yamane 2008) and upon recombination of nonidentical DNA molecules (Surtees *et al.* 2004). If the damaged or mismatched DNA is not repaired, and a new round of replication is initiated, the mutation becomes stably incorporated into the genome.

Lynch syndrome is a prevalent hereditary cancer syndrome caused by defects in DNA mismatch repair (Lynch *et al.* 2009). Individuals with Lynch syndrome are typically heterozygous for either *MSH2* or *MLH1*, core components of DNA mismatch repair (Silva *et al.* 2009). As part of the disease process, the sole wild-type copy of the mismatch repair gene becomes inactivated, and a cell then begins to accumulate mutations at an accelerated rate, often leading to tumor formation (Boland 2012; Colas *et al.* 2012). A distinguishing feature of most mismatch repair defective tumors is the presence of microsatellite instability (Shah *et al.* 2010a). Microsatellites are composed of repetitive sequences with 1−10 nucleotides as the repeat unit (reviewed in Bhargava and Fuentes 2010; Gemayel *et al.* 2010). Microsatellite instability is a consequence of unrepaired slippage events during DNA replication of these repeat regions (Levinson and Gutman 1987) and is confirmed when length of the microsatellite loci from an individual’s tumor differs significantly from the same loci in healthy cells (Lynch *et al.* 2009). In addition to frequently displaying microsatellite instability, mismatch repair defective tumors tend to be diploid on a gross chromosomal level, as opposed to the more typical aneuploidy observed in other cancers (Oki *et al.* 2012).

Since the discovery of the link between mismatch repair and Lynch syndrome, many germline and somatic mutations have been identified in mismatch repair genes (de la Chapelle 2004). Approximately 20% of these mutations are missense variants, resulting in a single amino acid substitution in the mismatch repair protein (de la Chapelle 2004). Our previous characterization of these missense variants has provided insights into the molecular defects associated with Lynch syndrome cancers (Gammie *et al.* 2007). In this work, we analyzed clinically significant missense variants of *MSH2* along with the *msh2* null in yeast to characterize the genomic signature associated with Lynch syndrome.

Our current understanding of the effects of mismatch repair deficiency on genome stability is derived mainly from analyses using reporter genes in organisms ranging from bacterial to human systems (reviewed in Aquilina and Bignami 2001). The types of reporters include those that assay single-base substitutions and/or microsatellite instability of mono-, di-, tri-, and larger nucleotide repeats (Hawk *et al.* 2005; Henderson and Petes 1992; Marsischky *et al.* 1996; Tran *et al.* 1997). These reporters are typically expressed episomally or integrated into the genome at select loci. Although informative, reporter constructs do not reveal the full spectrum of possible mutations, nor do they capture mutational variability associated with genomic architecture, sequence contexts, or processes such as replication and transcription.

The mutation accumulation assay provides an alternative to reporter assays. In a mutation accumulation assay, the population is propagated through recurrent single-cell bottlenecks, thus mitigating the effect of selection and allowing mutations (other than lethal mutations) to accumulate as if they were neutral. Sequencing the end point of a lineage reveals the number, positions, and identities of accumulated mutations. In this work, we passaged mismatch repair defective haploid yeast cells over hundreds of generations with recurrent bottlenecks and determined the mutation rates, spectra, and genome-wide distributions of mutations by using whole-genome sequencing. We find that mismatch repair deficient strains accumulate ~1 mutation per genome per generation (corresponding to a ~200- to 300-fold increase in mutation rate relative to wild type). Because the mutation accumulation assay queries many types of mutation events and contexts simultaneously, it not only produces a more accurate estimate of the per-genome per-generation mutation rate, but also allows one to determine how the mutation rate is influenced by sequence-specific features and genomic context. We find that mutations occurred randomly across the genome, with no chromosomal, gene, or replication timing biases; however, mismatch repair defective cells do display a distinctive mutational signature, with deletions at homopolymeric runs representing the primary mutational event. We find that microsatellite instability increases with repeat length and that microsatellites adjacent to other repeats are more mutable. Overall, these data provide insight into the oncogenic process and should aid in the identification of the likely drivers of tumor formation in cancers displaying microsatellite instability.

## Materials and Methods

### Microbial and molecular techniques

Microbial manipulations were conducted according to previously published procedures (Ausubel *et al.* 1994; Burke *et al.* 2000). Molecular methods were performed with the use of standard protocols (Ausubel *et al.* 1994). Plasmid DNA extractions were performed using the Qiagen procedure (QIAGEN Inc., Valencia, CA). Primers were synthesized by Integrated DNA Technologies Inc. (Coralville, IA). Restriction endonuclease digestions and polymerase chain reaction (PCR) were performed using the enzyme manufacturer recommended reaction conditions (New England Biolabs, Beverly, MA).

### Strains and plasmids

XL2-Blue (Stratagene, La Jolla, CA) bacterial cells were used for plasmid propagation. The salient features of the plasmids used in this work are listed in the Supporting Information, Table S1). The *msh2* missense mutations encoded on centromere-based plasmids were generated as described previously (Gammie *et al.* 2007). The *msh2* knockout strain AGY1079 (*MAT*α *msh2*::*URA3hom3-10 ade2-1 trp1-1 ura3-1 leu2-3,112 his3-11,15*) and a wild-type strain from the same cross AGY1100 (*MAT***a** *hom3-10 ade2-1 trp1-1 ura3-1 leu2-3,112*) were derived from W303. The strains were confirmed to be wild type at the *RAD5* locus by PCR and at the *CAN1* locus by canavanine resistance assays.

### Qualitative mismatch repair and fluctuation assays

Qualitative mismatch repair assays as described previously (Gammie *et al.* 2007). Canavanine resistance was selected for using plates supplemented with 60 μg/mL canavanine (Sigma-Aldrich, St. Louis, MO). Luria-Delbrück fluctuation assays, used to determine the rates of loss of function of *CAN1* were performed as described previously (Lang and Murray 2008). Mutation rates were calculated using both the Luria-Delbrück P_0_ method (Luria and Delbrück 1943) and the MSS maximum-likelihood method (Sarkar *et al.* 1992).

### Mutation accumulation

The *msh2* knockout strain was transformed with the plasmids listed in Table S1 and propagated in synthetic medium lacking histidine to select for the plasmids. A single colony from each transformation was selected to begin the mutation accumulation experiment. Strains were passaged on synthetic medium lacking histidine for ~170 generations with bottlenecks every ~21 generations (Figure S1). The bottlenecks were accomplished by picking a single colony and streaking for single colonies approximately every 2 d; the process was repeated eight times. Taking into account population expansion between the bottlenecks, we estimate an effective population size of approximately 10. The theory underlying the mutation accumulation assay is that all mutations other than lethal mutations accumulate as if neutral. If the population size were exactly one, this would be true; however, the population expansion between bottlenecks introduces the opportunity for selection. Given a rate of one mutation per cell division, the likelihood of losing a strongly deleterious mutation (0.1) is only 10% (see Figure S1 in Lynch *et al.* 2008).

### Sequencing

In preparation for sequencing, a single colony was selected and grown in 25 mL of yeast extract, peptone, dextrose medium supplemented with adenine (Burke *et al.* 2000) until saturation was achieved (24−40 hr). Genomic DNA preparations from yeast were as described previously (Burke *et al.* 2000) except the glass bead lysis step was accomplished with a Fastprep-24 instrument (MP Biomedicals LLC).Yeast genomic DNA was prepared for sequencing with the Illumina TruSeq DNA Sample Preparation kit with six indices for multiplexing. Whole-genome sequencing was performed at the Lewis-Sigler Institute for Integrative Genomics Core Sequencing Facility with an Illumina HiSequation 2000. Four lanes with six samples each were used. The ancestor samples were doubled to maximize coverage. Single end reads of 100 bp were performed giving from 50x to 300x coverage of each genome (Table S2).

### Sequencing data analysis

Each sequencing read was aligned to a draft yeast genome with BWA for Illumina version 1.2.2 (Li and Durbin 2009) using parameters listed in Table S3. Mutations were identified using Freebayes version 0.8.9.a, a Bayesian single-nucleotide polymorphism and short insertion/deletion (indel) caller (Garrison and Marth 2012) using parameters listed in Table S4. The default parameters for the BWA mapping and Freebayes mutation calling programs missed almost all (93%) of the insertion/deletion mutation. Using the parameters listed in Table S3 and Table S4 was essential for calling the insertions/deletions. BWA and Freebayes were implemented using the Galaxy user interface (Blankenberg *et al.* 2010; Giardine *et al.* 2005; Goecks *et al.* 2010).

The draft W303 genome is available upon request and was generated as follows. Three ancestral W303 strains, including the wild-type (AGY1100) and *msh2* (AGY1079) ancestors described in this study as well as a wild-type W303 strain from a different cross (G. Lang collection), each with >300x coverage, were used to identify common and unique polymorphisms when compared with the S288C genome as detailed previously. The common polymorphisms were applied to the S288C reference using the FastaAlternateReferenceMaker utility from the Genome Analysis Toolkit (McKenna *et al.* 2010), generating an updated reference. The sequence reads were mapped to this new reference, and common polymorphisms were again identified and applied to the reference. This was repeated for several iterations and resulted in a final list of polymorphisms, including 9657 single-base-pair substitutions and small insertion/deletions. Larger insertion/deletions or duplications were not identified.

We identified 14 unique polymorphisms in the *msh2*∆ ancestor not found in the other two W303 ancestors (see Table S5). Seven were intergenic or within an intron, the remaining were missense/nonsense or frameshift mutations in well-characterized genes that are not associated with mutator phenotypes. These findings support the conclusion that the *msh2*∆ was the only mutator allele present in the starting strain.

The mutations in passaged lines were identified by mapping to the draft W303 genome and comparing the called mutations from the lineages with the ancestor. *MSH2* chromosomally encoded wild-type passaged line was compared to the wild-type ancestor and the plasmid based lines were compared to their shared *msh2*∆ ancestor. Each unique mutation in the passaged strains was verified manually using Integrative Genomics Viewer (Robinson *et al.* 2011; Thorvaldsdottir *et al.* 2012). Only fixed mutations (*i.e.*, mutations in 100% of the reads) were scored. Thus, mutations arising during the few generations required for obtaining genomic DNA for sequencing were not scored because these mutations would not be present in all of the reads. Insertions/deletions are difficult to score because of inherent problems with PCR amplifications and sequencing of repeat regions. To score as an insertion/deletion, at least three reads must have traversed the entire repeat region for both the passaged line and the ancestor.

We identified 10 lineages with three common end-point single base substitutions and two insertion/deletion mutations not present in the *msh2*∆ ancestor. We reasoned that these common mutations were likely to represent mutations that arose during growth of the ancestral strain prior to transformation (Figure S1). To test this, for each of the five common mutations, using PCR we amplified and resequenced the region from the first time point of each lineage (frozen immediately after transformation). In all cases the common mutations were observed immediately after transformation, suggesting that these five mutations occurred during growth of the ancestral strain prior to the transformation of the plasmids. We, therefore, removed these mutations from subsequent analyses.

To assess mutation rates at microsatellites, an accurate count of the repeat number was required. Microsatellites in the draft W303 genome were identified using msatfinder (Thurston and Field 2005). Bedtools IntersectBed (Quinlan and Hall 2010) was used to find the number of reads that overlap a microsatellite region as well as non-repeating regions of varying length. Using R for Statistical Computing (http://www.r-project.org/) regions from chromosome XII (rDNA repeats) as well as regions with a read count ≥4x median were removed before plotting. R was also used to generate box plots of the number of reads that span the regions of each length, stratified by repeating or nonrepeating.

## Results

### DNA mismatch repair defective cells accumulate approximately 1 mutation per generation, ~200- to 300-fold greater than the wild-type rate

Until recently (Ma *et al.* 2012; Nishant *et al.* 2010; Zanders *et al.* 2010), obtaining estimates of the increase in mutation rate in mismatch repair defective cells depended solely on reporter genes. In this study, we calculated the mutation rates across the entire genome by using haploid wild-type and mismatch repair defective cells in a mutation accumulation assay over ~170 generations (Figure S1). We tested 16 clinically significant missense variants of *msh2* by expressing each from a centromere-based plasmid in an *msh2*∆ strain. The wild-type control was the *msh2*∆ strain containing the wild-type version of *MSH2* expressed from a centromere-based plasmid (CEN WT) and the *msh2*-null control was the *msh2*∆ strain with the empty plasmid vector. The mutation accumulation experiment also included a wild-type control in which *MSH2* was intact in the chromosome (genomic WT). After passaging, genomic DNA was prepared for whole-genome sequencing. The sequencing depth ranged from 50x to 300x coverage (Table S2). The mutations in each passaged strain were compared with the relevant ancestor (genomic WT, or the *msh2*-null ancestor). All mutations were manually verified as described in the *Materials and Methods*.

In this analysis (Table 1) and previously (Arlow *et al.* 2013; Gammie *et al.* 2007) we used the plasmid based controls to classify the missense variants into functional categories: null, intermediate, and wild type. In the current study, one missense mutant, *msh2-P689L*, was classified as a pseudo-wild type based on the fluctuation assays, whereas the remaining missense strains were indistinguishable from the null allele (Table 1). For the remainder of the paper, unless specifically indicated, we combined the mutations for the 16 *msh2*-null-like strains for increased statistical power. Three strains harbored rearranged plasmids in which the *MSH2* coding sequence was not intact (noted in Table 2). The rearrangement occurred early in the passaging and these variants were thus classified as true nulls for certain statistical tests.

**Table 1:** Classification of sequenced strains

Functional Domain	Relevant Genotype (*CEN*)	Class	Mutation Rate Can^r^*^a^*	Fold Induction Can^r^	n
	*msh2*Δ	Null	6.7 (6.3−7.0) × 10^−6^	8	930
	*MSH2 CEN*	CEN WT	8.0 (7.4−8.6) × 10^−7^	1	609
Structural integrity	*msh2-A618V*	Null	6.0 (5.2−6.8) × 10^−6^	7	144
	*msh2-R657G*	Null	6.2 (3.7−9.2) × 10^−6^	8	72
	*msh2-L183P*	Null	7.1 (6.1−8.1) ×10^−6^	9	144
	*msh2-C195Y*	Null	8.5 (7.2−9.9) × 10^−6^	11	72
	*msh2-C345F*	Null	6.8 (5.8−7.8) × 10^−6^	8	144
	*msh2-D621G*	Null	9.6 (8.0−11.4) × 10^−6^	12	72
	*msh2-P640T*	Null	9.1 (7.9−10.3) × 10^−6^	11	141
DNA binding	*msh2-R542L*	Null	6.3 (5.4−7.3) × 10^−6^	8	144
	*msh2-D524Y*	Null	4.8 (4.0−5.7) × 10^−6^	6	72
ATPase	*msh2-G688D*	Null	7.8 (6.8−8.8) × 10^−6^	10	144
	*msh2-G693R*	Null	3.8 (3.2−4.4) × 10^−6^	5	144
	*msh2-S695P*	Null	5.0 (4.3−5.7) × 10^−6^	6	144
	*msh2-S742F*	Null	6.6 (5.9−7.5) × 10^−6^	8	153
	*msh2-T743K*	Null	8.7 (7.5−9.9) × 10^−6^	11	144
	*msh2-G770R*	Null	5.5 (4.8−6.3) × 10^−6^	7	139
	*msh2-P689L*	Pseudo-WT	6.0 (4.9−7.2) × 10^−7^	1	144

a Confidence limits in parentheses. WT, wild type.

**Table 2:** Mutation rate based on mutation accumulation over ~170 generations

Functional Domain	Relevant Genotype	Single-Base Pair Substitutions	Insertions or Deletions	Mutation Rate Overall*^a^*	Fold Induction WT*^b^*
Genomic WT	*MSH2*	1	0	4.8 × 10^−10^	1
Null	*msh2*Δ	7	140	7.1 × 10^−08^	215
Structural integrity	*msh2-A618V*	8	109	5.7 × 10^−08^	171
	*msh2-R657G*	6	141	7.1 × 10^−08^	215
	*msh2-L183P*	7	143	7.2 × 10^−08^	220
	*msh2-C195Y**^c^*	15	158	8.4 × 10^−08^	253
	*msh2-C345F*	16	180	9.5 × 10^−08^	287
	*msh2-D621G**^c^*	12	144	7.5 × 10^−08^	228
	*msh2-P640T*	10	125	6.5 × 10^−08^	198
DNA binding	*msh2-R542L*	4	135	6.7 × 10^−08^	203
	*msh2-D524Y*	14	151	8.0 × 10^−08^	242
ATPase	*msh2-G688D*	15	139	7.4 × 10^−08^	225
	*msh2-G693R*	9	146	7.5 × 10^−08^	227
	*msh2-S695P**^c^*	14	159	8.4 × 10^−08^	253
	*msh2-S742F*	9	156	8.0 × 10^−08^	242
	*msh2-T743K*	5	147	7.3 × 10^−08^	223
	*msh2-G770R*	7	147	7.4 × 10^−08^	225

a Mutations per base pair per generation.

b Fold induction compared with a previously published rate 3.3 × 10^−10^ (Lynch *et al.* 2008).

c Plasmid rearrangement, effectively a null.

In the *msh2*-null strains, we identified 158 base pair substitutions and 2318 insertion/deletion mutations across the 16 lineages. The average rate of mutation for the *msh2*-null strains was 7.4 × 10^−8^ mutations per base pair per generation (Table 2). This rate is two orders of magnitude greater than the estimate of 3 × 10^−10^ mutations per base pair per generation for wild-type yeast strains (Lynch *et al.* 2008; Nishant *et al.* 2010); the genomic wild-type strain accumulated only a single mutation over the 170 generations, consistent with a wild-type per-base pair per-generation mutation rate of ~10^−10^ mutations per base pair per generation. In the absence of mismatch repair, the mutation rate for single-base pair substitutions was 4.8 × 10^−9^ mutations per base pair per generation, and for insertions or deletions at mono-, di-, and trinucleotide repeats was 7.0 × 10^−8^ mutations per base pair per generation. Overall, this suggests a 225-fold increase over genomic wild-type in the number of mutations for mismatch repair defective cells, or ~1 mutation per genome per generation.

### In the absence of mismatch repair, mutation accumulation occurs randomly with respect to chromosomal position

**Figure 1:**
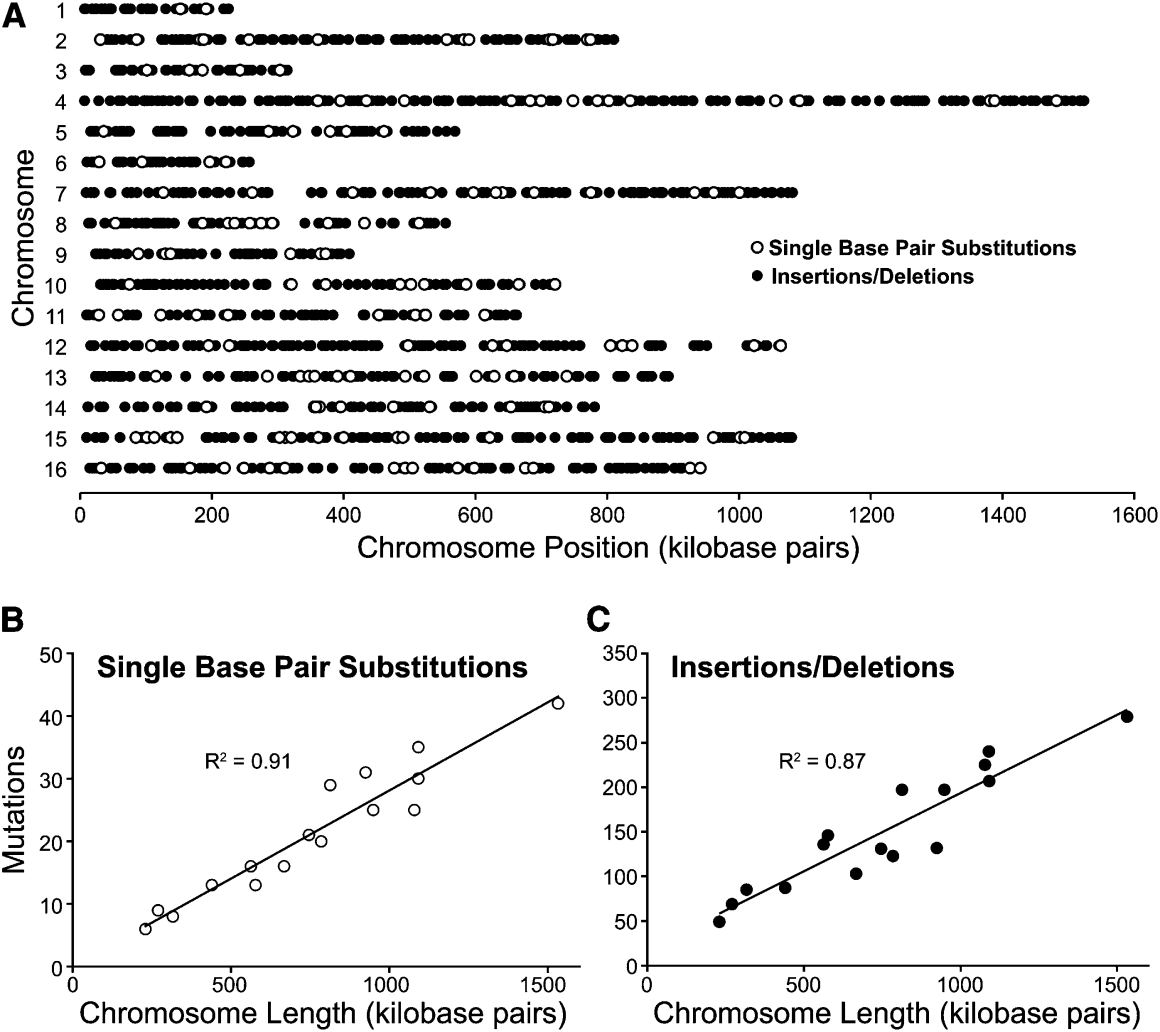
Mutations in mismatch repair defective cells occur randomly across the genome. (A) Chromosomal distribution of mutations including the single base pair substitutions (open circles) and the insertions/deletion at mono-, di-, and trinucleotide microsatellites (filled circles) are shown at their chromosomal position for each of the 16 yeast chromosomes. Mutation number was plotted against chromosome size for single-base pair substitutions (B) and for insertions/deletions at microsatellites (C). Single-base substitutions in (B) represent data pooled from two independent mutation accumulation experiments. R^2^ values were generated in Microsoft Excel (Redmond, WA) and are indicated on the graphs.

Previous experimental and comparative genomic analyses in yeast showed that there are mutational differences with respect to the chromosomal context (Hawk *et al.* 2005; Ito-Harashima *et al.* 2002) and replication timing (Agier and Fischer 2012; Lang and Murray 2011). Examining the mutations across the entire genome allowed us to determine if there were any position effects that might relate to chromosomal structure or replication timing. We determined that both single base pair substitutions and insertions or deletions at repeats occurred randomly across the genome (Figure 1A). In keeping with this, the number of single base pair substitutions (Figure 1B) and insertions/deletions (Figure 1C) per chromosome correlated with chromosome size (R^2^ = 0.91 and 0.87, respectively).

Although the mutation positions were random at a gross chromosomal level, we wanted to determine if they were in regions that have been associated with greater mutation rates such as late replicating portions of the genome. By binning the genome by replication timing (Raghuraman *et al.* 2001) at 10-min intervals and calculating the mutation rate for each bin, we fail to find a significant correlation between replicating timing and the mutation rate (*P* = 0.31, χ^2^).

Because these experiments did not depend on reporter genes, we analyzed whether there was any relationship between mutation position and coding sequences. We found that the single base pair substitutions occurred mostly in coding regions (72%). This number is in contrast to the insertions/deletion mutations that were more likely to be in noncoding regions than in coding sequences (14%), reflecting the composition of the yeast genome. Approximately 74% of the yeast genome is comprised of coding sequences (Cherry *et al.* 1997) consistent with the distribution of single base pair substitutions. Additionally, only 10–20% of the microsatellite DNA, including mono-, di-, and trinucleotides, is found in eukaryotic coding sequences (Li *et al.* 2004), similarly reflecting the distribution of insertions/deletion mutations we identified. Taken together, these data suggest that any mutational bias associated with chromosome structure, gene organization, or replication timing is diminished in the absence of mismatch repair.

### Insertion/deletion loop repair is the predominating mismatch repair role required During passaging of cells over 170 generations

Measuring the frequency for the entire spectrum of mutations at endogenous loci in parallel was not possible until recently. Here we report the concurrent measurement of mutation frequency of single base pair substitutions as well as insertions/deletions at mono-, di-, and trinucleotide repeats (Table 3). For the remainder of this work, we will maintain a distinction between single nucleotide microsatellites (homopolymeric runs) and larger di-, tri-, and tetranucleotide microsatellites. We find that the mutation frequency spectrum for mismatch repair defective cells included deletions/insertions at homopolymers (87.7%) and at di- and trinucleotide microsatellites (5.9%), as well as transitions (4.5%) and transversions (1.9%).

**Table 3:** Summary of genome-wide mutations in mismatch defective cells

Mismatch Type	Mutation	Number*^a^*	% Total
Single-base indel*^b^*	Deletions at homopolymers	2011	81.2
	Insertions at homopolymers	161	6.5
Subtotal		2175	87.7
Single base substitution	Transitions	112	4.5
	Transversions	46	1.9
Subtotal		158	6.4
Larger indel*^a^*	Insertions at microsatellites	86	3.5
	Deletions at microsatellites	60	2.4
Subtotal		146	5.9

a Data from all strains defined and *msh2* null.

b Indel, insertion/deletion, only two indels were not at homopolymers or larger microsatellites.

### In the absence of mismatch repair, the mutation rate at homopolymeric runs and microsatellites increases nonlinearly with repeat length

**Figure 2:**
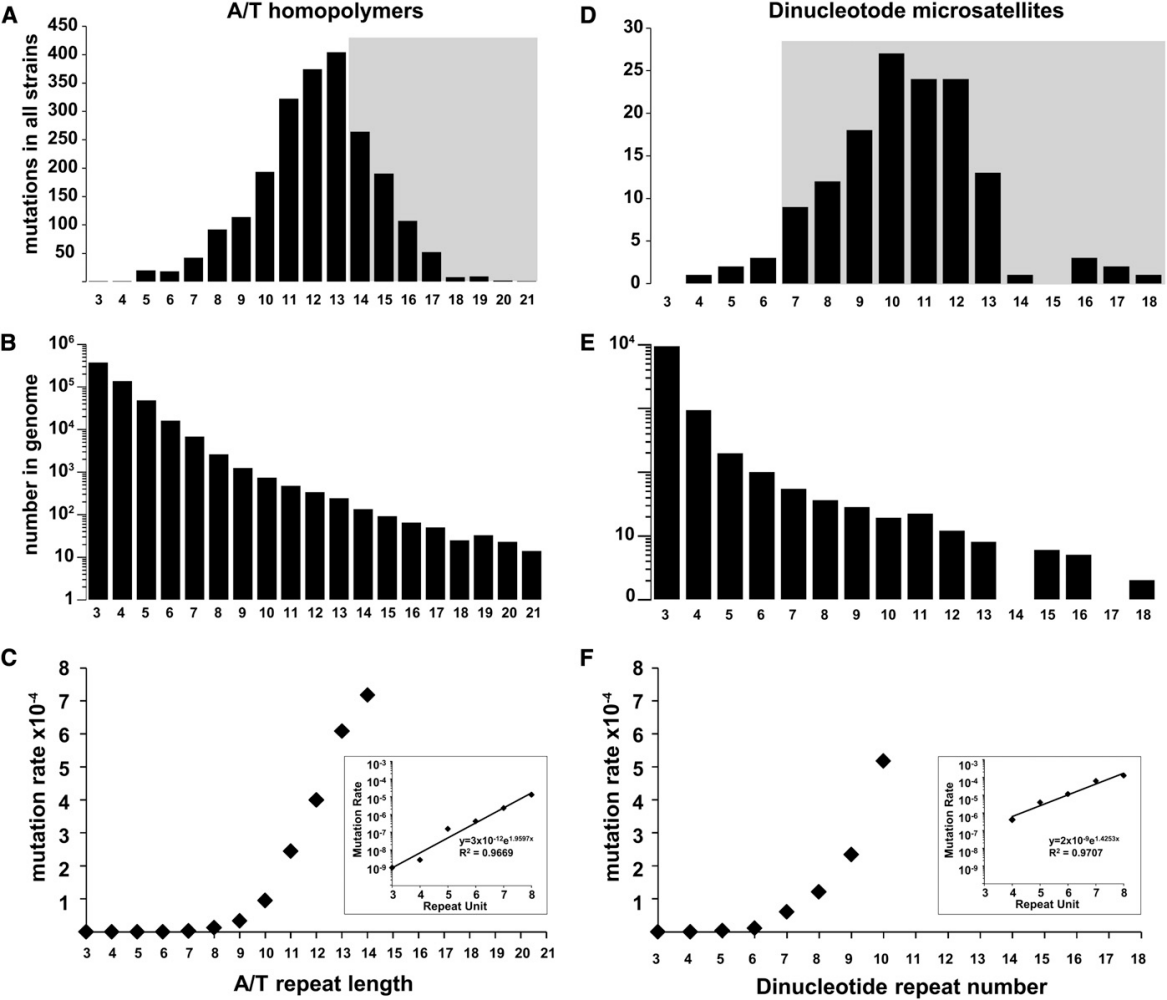
Mutation rate increases with microsatellite repeat length. The number of insertion/deletion mutations identified at A/T homopolymeric repeats (A), or dinucleotide microsatellites (D) are plotted according to repeat length. Shaded areas indicate that the numbers might be an underrepresentation because of the decreased ability to detect insertions or deletions at long repeats. The number of A/T homopolymers (B) or dinucleotide microsatellites (E) in the yeast genome (y-axis) is plotted according to repeat length (x-axis) on semi-log graphs. The mutation rate (mutation per repeat per generation) for homopolymers (C) or dinucleotide microsatellites (F) are plotted according to repeat unit. The exponential increase in mutation rate from 3 to 8 repeat units is plotted on semi-log graphs in enclosed panels. Formulas and R^2^ values were generated in Microsoft Excel.

Previous work showed that the mutation rate at microsatellites increased with repeat unit length (Tran *et al.* 1997; Wierdl *et al.* 1997). In this study, we compared the rates of mutation at endogenous microsatellite loci and over hundreds of generations using multiple strains in parallel. We confirmed that the number of mutations increased with repeat length (Figure 2, A and D) at a much higher frequency than was expected from the occurrence of such repeats in the genome (Figure 2, B and E, note the log scale). The strong length dependence on instability is evident with each additional repeat unit resulting in a progressive fourfold and sevenfold increase in sequence instability for homopolymers and larger microsatellites, respectively.

The mutation rate data for homopolymers and larger microsatellites revealed a striking, overall nonlinear increase in the mutation rate with repeat length (Figure 2, C and F). The mutation rates at homopolymers and dinucleotide microsatellites show an exponential increase with repeat unit until reaching a repeat unit of eight. For example, the rate of mutations per repeat per generation for (A/T)_n_ homopolymer runs ranged from 9.7 × 10^−10^ (repeat unit of three) to 1.3 × 10^−5^ (repeat unit of eight). For repeat units greater than nine, the observed increase in rate changed from exponential to linear (y = 0.0001x − 0.0012; R^2^ = 0.98). The same trends were also observed for (C/G)_n_ homopolymers, but with slightly greater mutation rates (~7-fold greater on average, not shown). The differences in rates at the two types of homopolymers have been observed previously (Gragg *et al.* 2002); however, in this study, the sample size for (C/G)_n_ homopolymers was significantly lower (n = 38 compared with n = 2134) and therefore the apparent differences in rates may be a consequence of the number of events measured. The trend from exponential to linear at repeat units greater than nine was also observed for dinucleotide microsatellites; however the data are less accurate beyond repeat units of seven because of the lower sample size.

The change in the rate increase from exponential to linear may have a biological explanation; however, we speculate that the rates are less accurate for longer repeats, because multiple sequencing reads must traverse the entire repeat to confidently call an insertion or deletion mutation. We performed an analysis of sequencing read counts that spanned entire repeats for all of the sequenced strains and found a significant drop with repeats greater than 13 bp regardless of the genome coverage (Figure S2). Therefore, our ability to detect an insertion/deletion mutation in repeats greater than or equal to 14 bp in length is diminished, leading to underestimates of the true mutation rate at these positions (gray shading in Figure 2, A and D).

The larger quantity of mutations at homopolymers, relative to dinucleotide repeats, does not result from a greater rate of mutation at homopolymers. In fact, for repeat units between five and seven the rate of mutation of homopolymers is ~20-fold less than that of dinucleotides of the same repeat unit. The greater number of observed mutations in (A/T)_n_ homopolymers simply reflects the relative abundance in the yeast genome (compare Figure 2, B and E).

### A mutational bias toward deletions at homopolymeric runs and insertions at certain microsatellites is observed in mismatch repair defective cells

When assaying for insertion/deletion events, some reporter loci influence the type of mutation because of reading frame constraints, the requirement for active transcription, the proximity and orientation with respect to origins of replication, and/or unusual chromatin structure. Mutation accumulation followed by genome-wide sequencing allows for the determination of any potential insertion/deletion bias at mono-, di-, and tri- microsatellites without the use of reporter loci.

Although the increase in mutation rate at homopolymers and dinucleotide microsatellites is similar when adjusted for repeat unit, we observed a difference in the types of mutations generated at these sites (Table 4). We find that (A/T)_n_ homopolymers suffer deletions at a high rate (93%, n = 2134, *P* < 10^−10^, χ^2^). The (C/G)_n_ repeats also have a bias toward deletions, but it is less pronounced (74%, n = 38, *P* = 3.5 × 10^−3^, χ^2^). The (GT/CA)_n_ dinucleotide microsatellite instability events show a trend toward deletions (65%, n = 17, *P* = 0.23, χ^2^), although this finding is not statistically significant. In contrast, (AT/TA)_n_ dinucleotide microsatellite instability shows a significant insertion bias (63%, n = 113, *P* = 6.4 × 10^−3^, χ^2^). Finally, the trinucleotide repeats show a slight tendency toward insertions (57%, n = 14); however, the number of events was not sufficient to for a statistical analysis to determine an insertion/deletion bias within each sequence type. In summary, the bias toward an insertion or deletion event is likely to be dependent on the composition of the repeat.

**Table 4:** Insertion/deletions at homopolymeric runs and larger microsatellites

	A/T	C/G	HPR Total	AT/TA	GT/CA	GA/CT	AAT/ TTA	AAC/ TTG	ATT/ TAA	ACG/ TGC	ATG/ TAC	di/tri MS Total
Total	2134	38	2172	113	17	2	2	4	3	1	4	154
Insertion	151	10	161 (7%)	71	6	1	1	1	3	0	3	94 (61%)
Deletion	1983	28	2011 (93%)	42	11	1	1	3	0	1	1	60 (39%)

HPR, homopolymeric run; di/tri MS, di- and tri- nucleotide microsatellites.

### DNA regions with a greater density of repeats are more mutable in mismatch repair defective cells

**Figure 3:**
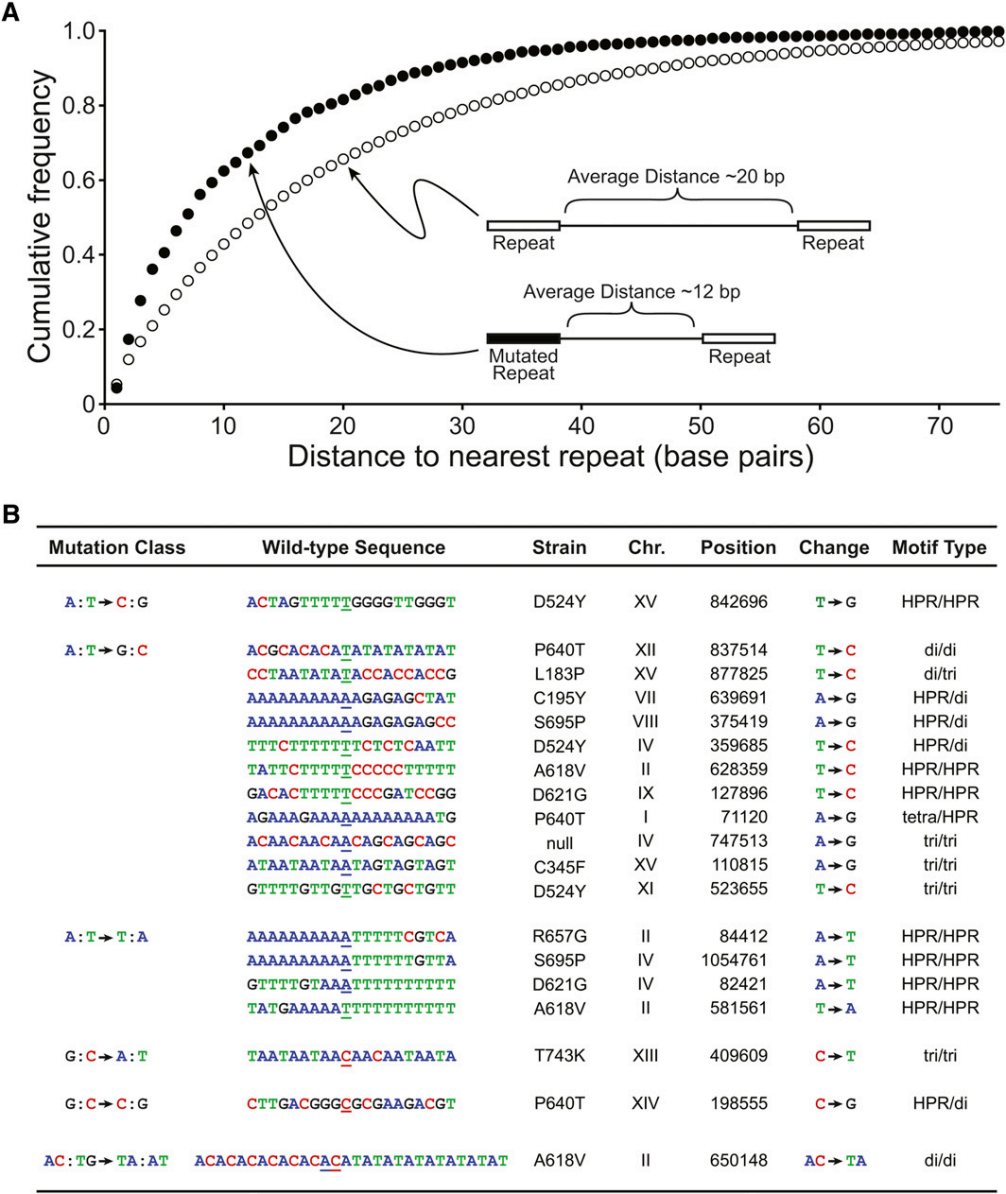
Microsatellites proximal to other repeats are more mutable. (A) The cumulative frequency plots for microsatellites sorted according to the distance to the nearest neighboring repeat for the whole genome (open circles) or for the mutated regions (closed circles) are shown. MATLAB (MathWorks, Inc.) kstest2, Kolmogorov-Smirnov comparison of two data sets, was used to determine the p value, *P* = 2.8 × 10^−6^. The schematic diagram provides an illustration of the relative distance between repeats for the whole genome compared with the mutated microsatellites and the nearest neighboring repeat for a particular point on the graph. (B) The table lists single base substitutions found in regions with immediately adjacent repeats, including homopolymeric runs (HPR), dinucleotide (di), trinucleotide (tri), and tetranucleotide (tetra) microsatellites. The nucleotide sequence is shown and the wild-type base that is mutated in the experimental strain is underlined. The nucleotide change is indicated as is the mutational class. The chromosome position is given for the W303 draft genome (available upon request).

Although no gross chromosomal mutational hotspots were identified, we observed that regions with a higher density of repeats were more mutable. We used motif-searching algorithms and observed that the mutated mono-, di-, or tri nucleotide repeat loci were often found in close proximity to other repeats. For example, we find that 28% of the mutated repeats are within 3 bp of the next repeat in the genome and 51% are 7 bp from the most adjacent repeat. To determine if this was statistically significant we sorted the loci according to the closest adjacent repeat and plotted the cumulative percentages of all genomic repeat loci and the mutated repeat loci (Figure 3A). The plot illustrates the differences between the distributions. Using a Kolmogorov-Smirnov comparison of two data sets we find that there is a statistical difference (*P* = 2.8 × 10^−6^), confirming that repeats are more mutable if there is a proximal repeat. This finding is in agreement with comparative genomic analyses (McDonald *et al.* 2011) and with genome-wide sequencing of the accumulated mutations in mismatch repair defective yeast cells (Ma *et al.* 2012).

We also used motif finding algorithms to find potential consensus site for single base pair substitutions. One of the most striking motifs represented regions with adjoining repeat sequences (Figure 3B). Based on the elevated mutation rates of mono-, di-, and trinucleotide microsatellites (Figure 2) and on the increased mutability if the repeats are proximal (Figure 3, A and B), we speculate that certain single base pair substitutions might, in fact, reflect double slippage events rather than DNA polymerase base substitution errors.

### The mutation spectra of certain *msh2* alleles differ from the *msh2* null- and wild-type cells

As mentioned previously, we find that the mutation frequency spectrum for the combined mismatch repair defective cells included ~6% single base pair substitutions, as well as deletions/insertions ~88% at homopolymers and ~6% at di- and trinucleotide microsatellites. We tested whether any of the strains expressing the *msh2* alleles had a different mutation spectrum when compared to the null. Although the missense mutant spectra were not significantly different from the null spectrum (all *P* > 0.01), five mutants had slightly altered ratios (*P* < 0.05, see Table S6). The differences were primarily accounted for by more insertion/deletions at di- and tri nucleotide repeats.

**Figure 4:**
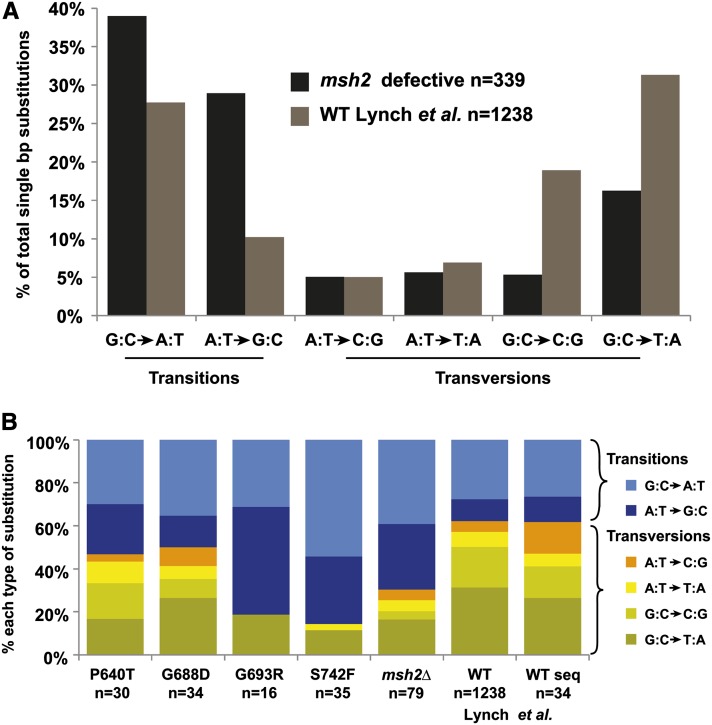
Single-base substitution signature for mismatch repair defective cells. (A) The percentages of each class of single-base substitutions are shown for the pooled mismatch repair defective cells (*msh2*) and the wild-type reporter construct data (Kunz *et al.* 1998; Lang and Murray 2008; Ohnishi *et al.* 2004) compiled by Lynch *et al.* (*i.e.*, WT Lynch *et al.*) (Lynch *et al.* 2008). Transitions and transversions are indicated. The sample size for each strain is given (n). (B) The single-base-pair substitution signatures for the strains completely lacking *msh2* function (*msh2*∆), for the Lynch *et al.* (2008) wild-type sequencing data (WT seq Lynch *et al*.) and the wild-type reporter data (WT Lynch *et al.*) (Kunz *et al.* 1998; Lang and Murray 2008; Ohnishi *et al.* 2004) from panel (A) and for strains expressing missense variants of *msh2* indicated on the graph as the amino acid substitution (*e.g.*, P640T, proline at codon 640 in the yeast coding sequence is mutated to a threonine). Only signatures that were statistically different (*P* < 0.01) from the *msh2*∆ signature using the Fisher exact test (MATLAB script, Guangdi, © 2009) are shown. All but P640L missense substitutions fall in the ATPase domain of Msh2. The sample size for each strain is given (n). Single-base substitutions in this figure represents data pooled from two independent mutation accumulation experiments.

Mismatch repair defective cells have historically been associated with microsatellite instability, but the distinctive mutational spectrum for single base substitutions was not well established. Because the number of observed base-pair substitutions is low (163), we bolstered this data with a replicate mutation accumulation experiment through 200 generations (A. Gammie, unpublished data). Analysis of the pooled data set revealed that there is a characteristic signature for single-base pair substitutions in mismatch repair defective cells. Figure 4A shows the differences between the reported signature for wild-type (Lynch *et al.* 2008 and references therein) compared with the mismatch repair defective one from our analysis. Unlike wild-type yeast cells, where transversions predominate with G:C > T:A being the most common, mismatch repair defective cells accumulate more transition mutations, particularly G:C > A:T substitutions.

We tested whether any of the 16 *msh2* missense variants displayed a unique spectrum of base-pair substitutions when compared to wild-type or the *msh2* null. As noted previously and in Table 2, three strains suffered plasmid rearrangements early in the passaging and were subsequently treated as true nulls. The single-base pair mutation distribution from these strains were combined with the null (*msh2∆* + vector) and the spectrum was found to be statistically different when compared to the reported values for wild-type using χ^2^ analysis (*P* = 4.82 × 10^−8^) and Fisher exact tests (*P* = 0.01). Several of the missense variants showed differences (*P* ≤ 0.01) from the null set using the Fisher Exact test (Figure 4B). On the basis of our previous characterization of these variants (Gammie *et al.* 2007), we observed that these particular missense alleles express detectable quantities of the defective protein with alterations that mostly affected the ATPase domain (G688D, G693R, S742F; Figure 4B). We found that removal of the strains with statistical differences (*P* < 0.01) from the aggregate data set did not significantly affect our calculations of mutation rates or mutational spectra.

## Discussion

### The mutation rate in the absence of mismatch repair

Mutations in mismatch repair proteins, among the strongest elevators of mutation rate (Huang *et al.* 2003), are commonly observed in long-term evolution experiments as well as in commensal and pathogenic strains (LeClerc *et al.* 1996; Matic *et al.* 1997; Oliver *et al.* 2000) and are associated with Lynch syndrome, a heritable predisposition to cancer (reviewed in da Silva *et al.* 2009). Yet, despite the importance of the mismatch repair mechanism, we have an incomplete understanding of the mutation rate and spectra associated with defects in mismatch repair. Previous calculations placed the fold-increase in mutation rate for mismatch repair defective cells between 10^1^ and 10^4^ (reviewed in Kunkel and Erie 2005). The large range is attributable to the variable mutability of different sequences. For example, homopolymeric runs have been shown to have as high as a 5 × 10^4^-fold increase in mutation rates in mismatch repair defective yeast (Tran *et al.* 1997); whereas the *CAN1* locus shows only a 40-fold elevation (Marsischky *et al.* 1996). Traditionally, mutation rate estimates are made at individual reporter loci. Here we report whole genome sequencing of 16 mutation accumulation lines containing mismatch repair defective alleles of *msh2*. By assaying the accumulation of mutations genome-wide, this method averages over differences at individual loci to provide an accurate estimate of the per-genome per-generation mutation rate in mismatch repair defective cells. We find that the average mutation rate for mismatch repair defective cells is 7.5 × 10^−8^ mutations per base pair per generation, corresponding to approximately one mutation per genome per generation. This is consistent with a recent mutation accumulation experiment using a mismatch repair deficient, temperature-sensitive mutation in *mlh1* (Zanders *et al.* 2010). Our true wild-type line, in contrast, accumulated only a single mutation over the 170 generations of growth, consistent with previous estimates of the wild-type per-base pair, per-generation mutation rate on the order of 10^−10^, or one mutation ever few hundred generations (Drake 1991; Lang and Murray 2008; Lynch *et al.* 2008).

### Why chromosomal and replication timing effects disappear in mismatch repair defective cells

Previous work has demonstrated a correlation between mutation rate and replication timing (Agier and Fischer 2012; Lang and Murray 2011). We find, however, no correlation between mutation rate and replication timing in mismatch repair deficient lines. Our data are consistent with a random distribution of mutations across the genome as would be expected if mismatch repair has an equal opportunity to correct replication errors across the genome. This is supported by the previous observation that removing mismatch repair decreases the position effects on mutation rate (Hawk *et al.* 2005).

A previous study has implicated the action of translesion polymerases on late-replicating regions as a possible mechanism underlying the correlation between mutation rate and replication timing in mismatch repair proficient cells (Lang and Murray 2008). If mismatch repair were capable of correcting errors introduced by translesion polymerases, one would expect the absence of mismatch repair to exacerbate the correlation between replication timing and mutation rate. We do not see this, nor do we observe any mutations with the characteristic spectra of translesion polymerases. Overall the genome-wide distribution and spectra of mutations in mismatch repair deficient lines is consistent with mismatch repair correcting errors by the replicative, but not translesion polymerases.

### The mutation rate at homopolymeric runs and microsatellite sequences increases with length in the absence of mismatch repair

The mismatch repair machinery is responsible for binding and repairing insertion/deletion loops that go undetected by the DNA polymerase proof-reading function (reviewed in Hsieh and Yamane 2008). Interesting, when the repeat length of microsatellites surpasses 8−10 base pairs, the insertion/deletion loop is postulated to have the capacity to be propagated to a region outside the proof-reading domain of the DNA polymerase (reviewed in Bebenek *et al.* 2008; Garcia-Diaz and Kunkel 2006). The data presented in this paper show that in the absence of mismatch repair, the mutation rate increases exponentially with repeat length for both homopolymeric runs and larger microsatellites and switches to a linear increase as the repeat unit surpasses eight. If the threshold model is correct, there is an increased need for DNA mismatch repair to capture the unrepaired insertion/deletion loops as the microsatellite increases in length. This model, in part, explains the wide range of estimates for the effect of mismatch repair on mutation rate based on individual reporter loci.

Previously, several groups have attempted to determine in yeast whether a threshold exists, above which the repeats are unstable, and below which the mutability is indistinguishable from the background mutation (Pupko and Graur 1999; Rose and Falush 1998). We find mutations in homopolymeric runs as small as four nucleotides and mutations in microsatellites as small as three repeat units, or six nucleotides. Our findings that small repeats are mutable in the absence of mismatch repair are consistent with data from reporter constructs using homopolymeric repeats (Marsischky *et al.* 1996; Tran *et al.* 1997). Taken together, the data suggest that, if a threshold exists for increased mutability of homopolymers and microsatellites in the absence of mismatch repair, it is small.

### Model for insertion-deletion biases at microsatellites

Insertion/deletion mutations at microsatellites are thought to occur as a consequence of unrepaired DNA polymerase “slippage” events (Levinson and Gutman 1987). The genome-wide insertion/deletion mutation results in this work are in best agreement with previous *in vivo* reporter assays that did not bias the mutational event with reading frame constraints. These previous analyses revealed that in the absence of *MSH2*, homopolymers (Denver *et al.* 2005; Gragg *et al.* 2002; Marsischky *et al.* 1996) and (GT/CA)_n_ di-nucleotide microsatellites (Hawk *et al.* 2005) are more likely to suffer a single unit deletion. We speculate that the deletion bias is likely to be a consequence of DNA polymerase errors. Specifically, compelling crystal structure data revealed examples of DNA polymerase bound to DNA containing a single nucleotide deletion loop where the unpaired base is in the template strand (Bebenek *et al.* 2008; Garcia-Diaz *et al.* 2006). If such events were to go unrepaired *in vivo*, the newly synthesized strand would have a single nucleotide deletion. In addition, the (GT/CA)_n_ di-nucleotide deletion bias was observed *in vitro* with purified yeast replicative DNA polymerases using a gap filling assay (Abdulovic *et al.* 2011). Thus, DNA polymerase errors could account for the deletion bias at mono- and certain dinucleotide microsatellites.

In contrast, we observed an insertion bias at (AT/TA)_n_ di-nucleotides as well as some trinucleotide microsatellites. The bias toward insertion mutations at these sites might be attributed to the fact that most microsatellites have the capacity to form stable, complex non-B DNA structures *in vitro* (Kelkar *et al.* 2010; Richard *et al.* 2008). In some cases the secondary structure−forming microsatellites have been shown to inhibit DNA polymerase (Baran *et al.* 1991; Shah *et al.* 2010b). Although proving that such structures form *in vivo* is difficult, microsatellites are often sites of chromosome fragility, a phenomenon typically attributed to secondary structure formation and replication fork collapse (reviewed in Freudenreich 2007; Fungtammasan *et al.* 2012). We hypothesize that the formation of certain structures at microsatellites may cause increased pausing or switching of the DNA polymerase, thereby increasing the likelihood of the newly synthesized strand to become misaligned with the template. To fit the data, the (AT/TA)_n_ misalignment would have to occur with a bias toward slipping “back” one unit such that when the polymerase restarts, an extra unit will be introduced in the newly synthesized strand.

### Model for mutability of a microsatellite proximal to another repeat

In this work, we demonstrate that in the absence of mismatch repair, microsatellite repeats with proximal repeats are more likely to be mutated. This finding is in keeping with recent work describing mutational hot spots among clustered homopolymeric sequences (Ma *et al.* 2012). Additionally, comparative genomics suggests that the presence of a repeat increases the mutability of the region (McDonald *et al.* 2011). Several explanations exist for the increased mutability of repeats with proximal repeats, including the possibility of altered chromatin or transcriptional activity, or decreased replication efficiency (Ma *et al.* 2012; McDonald *et al.* 2011).

As mentioned previously, microsatellite repeats have the capacity to form an array of non-B DNA structures that decrease the fidelity of the polymerase (reviewed in Richard *et al.* 2008). Proximal repeats have the capacity to produce complex structural regions. For example, a well-documented chromosomal fragility site depends on an (AT/TA)_24_ dinucleotide repeat as well as a proximal (A/T)_19-28_ homopolymeric repeat for the formation of a replication fork inhibiting (AT/TA)_n_ cruciform (Shah *et al.* 2010b; Zhang and Freudenreich 2007). Additionally, parent-child analyses revealed that microsatellites with proximal repeats were more likely to be mutated (Dupuy *et al.* 2004; Eckert and Hile 2009). Finally, recent work demonstrated that a triplet repeat region inhibits the function of mismatch repair (Lujan *et al.* 2012). Taken together, we predict that the more complex secondary structures found at proximal repeats will increase the likelihood of DNA polymerase stalling or switching. At least two subsequent fates could account for an increase of insertion/deletions. First, the template and newly synthesized strand could misalign with the bulge outside of the DNA polymerase proof-reading domain. Second, if a lower-fidelity polymerase is installed at the paused replisome, the chances of an adjacent repeat or single base pairs in the vicinity becoming mutated would increase (McDonald *et al.* 2011). We further predict that mismatch repair function is not likely to be associated with error-prone polymerases and this could explain why some repeat regions might appear to inhibit mismatch repair.

### The most common mutations in mismatch repair defective tumors are likely to be insertion/deletions at homopolymeric runs

On the basis of the mutational signature we observed in yeast we predict that ~90% of the mutational events in a mismatch repair defective tumor will be single-base insertion/deletions within homopolymers, particularly those with proximal repeats. This prediction is based on the observations that humans and yeast are remarkably similar with respect to (1) the percentage of total microsatellite DNA (~3% in humans and ~4% in yeast; Lim *et al.* 2004; Subramanian *et al.* 2003), (2) the density of microsatellites (Richard *et al.* 2008), and (3) homopolymer to larger microsatellite ratio (Lim *et al.* 2004; Richard *et al.* 2008).

Interestingly, the redundancy of MutSα (Msh2/Msh6) and MutSβ (Msh2/Msh3) in recognizing a single-nucleotide insertion/deletion loop at homopolymeric runs (Acharya *et al.* 1996; Marsischky *et al.* 1996; Palombo *et al.* 1996; Umar *et al.* 1998) ensures that the most common mismatch generated during replication is likely to be identified and repaired. In keeping with this, tumor formation rarely arises as a consequence of loss of only Msh6 or Msh3 (de la Chapelle 2004). It will be of interest to determine whether the entire panel of rare *MSH6* Lynch Syndrome alleles confers a dominant negative function as has been previously reported for a variant of *MSH6* (Geng *et al.* 2012).

Given the mismatch repair deficiency mutation spectrum, we further predict that the drivers of tumor formation are likely to be genes that contain homopolymers with proximal repeats. Homopolymers and microsatellites represent unique challenges for whole genome sequencing algorithms designed to call mutations, resulting in a lower efficiency of confidently finding insertion/deletion mutations. For this reason, the candidate gene approaches are still commonly used when trying to determine cancer drivers in mutator tumor cells (The Cancer Genome Network 2012). Candidate cancer drivers encoding homopolymeric or larger microsatellite repeats have been extensively examined in mutator tumor cell lines; for example many potential drivers with homopolymeric runs, such as TGFBRII, are found to be frequently mutated in mismatch repair defective tumors (reviewed in Kim *et al.* 2010; Li *et al.* 2004; Shah *et al.* 2010a). Challenges in identifying true drivers in tumors with a high rate of mutation still remain because it is difficult to determine if an identified mutation was causative or simply a consequence of the repair defect. Additionally, finding novel tumor drivers is difficult because of the difficulty of whole genome sequencing in calling mutations at homopolymers and microsatellites. Going forward, computational approaches should allow for the detection of novel potential drivers based on the mutability of repeats with proximal repeats.

In this study, we have shown that the combination of mutation accumulation assays and next-generation sequencing is a powerful general method for revealing the genome-wide rate, spectra, and distribution of mutations in lines harboring Lynch Syndrome associated variants of the mismatch repair protein, Msh2. These data provide mechanistic insight into the mutagenic processes in the absence of mismatch repair and has potential as a tool for identifying target loci that contribute to the progression of this disease.

## Supplementary Material

Supporting Information
