# Supplementary material for: Mutation Rates, Spectra, and Genome-Wide Distribution of Spontaneous Mutations in Mismatch Repair Deficient Yeast
Source: G3 (Bethesda). 2013 Sep 1;3(9):1453–65. doi: 10.1534/g3.113.006429 (PMC3755907; doi:10.1534/g3.113.006429)
Supplement: Supporting Information [file supp_3_9_1453__index.html]

Mutation Rates, Spectra, and Genome-Wide Distribution of Spontaneous Mutations in Mismatch Repair Deficient Yeast — Supporting Information 

# Mutation Rates, Spectra, and Genome-Wide Distribution of Spontaneous Mutations in Mismatch Repair Deficient Yeast

## Supporting Information for Lang, Parsons, and Gammie, 2013

**Files in this Data Supplement:**

- Supporting Information - Figures S1-S2, Tables S1-S6, and Supporting References (PDF, 879 KB)
- Figure S1 - Schematic of Experimental Design (PDF, 312 KB)
- Figure S2 - Spanning Read Counts for Repeats (PDF, 410 KB)
- Table S1 - Plasmids used in the study (PDF, 244 KB)
- Table S2 - Sequencing Coverage (PDF, 277 KB)
- Table S3 - Parameters for Mapping with BWA for Illumina (PDF, 174 KB)
- Table S4 - Freebayes Parameters (PDF, 166 KB)
- Table S5 - Unique mutations in the *msh2*Δ ancestor (PDF, 331 KB)
- Table S6 - Mutation Spectra of Missense Variants (PDF, 295 KB)
- Supporting References - PDF, 239 KB
