# Supplementary material for: Mutation Rates, Spectra, and Genome-Wide Distribution of Spontaneous Mutations in Mismatch Repair Deficient Yeast
Source: G3 (Bethesda). 2013 Sep 1;3(9):1453–65. doi: 10.1534/g3.113.006429 (PMC3755907; doi:10.1534/g3.113.006429)
Supplement: Supporting Information [file supp_g3.113.006429_006429SI.pdf]

G3: Genes | Genomes | Genetics

Mutation rates, spectra, and genome-wide distribution of spontaneous mutations in mismatch repair deficient yeast

Gregory I. Lang*†, Lance Parsons*, and Alison E. Gammie§[1]
Lewis-Sigler Institute for Integrative Genomics* and Department of Molecular Biology§, Princeton University, Princeton NJ. † Present address: Department of Biological Sciences, Lehigh University, Bethlehem PA.

[1] Corresponding author, Mailing Address: Department of Molecular Biology, Princeton University, Princeton, NJ 08544-1014; Phone: (609) 258-6380, Fax: (609) 258-1975; E-mail: agammie@princeton.edu

DOI: 10.1534/g3.113.006429

Sequencing data are available through NCBI (SRA Study Accession Number SRP026313).

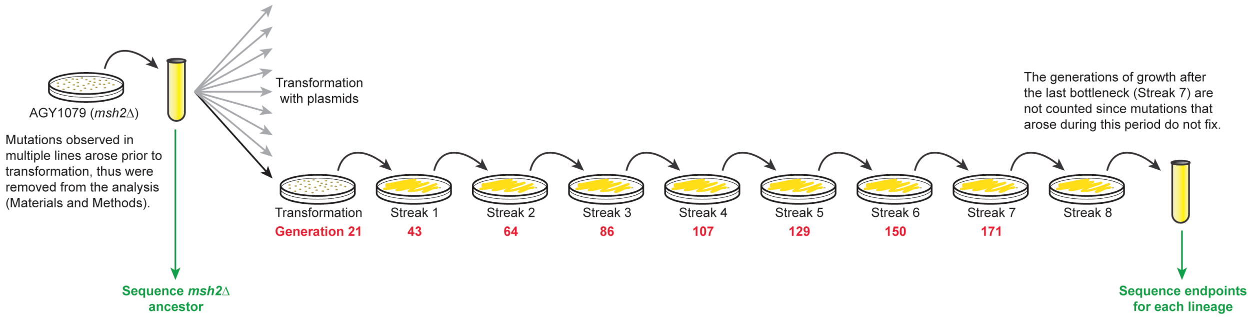

Figure S1: Schematic of Experimental Design. A single colony of AGY1079 was picked and transformed with *msh2*-containing plasmids. A single colony from each transformation was selected to begin the mutation accumulation experiment. Strains were passaged by streaking for single colonies every two days. We estimate ~21 generations of growth between bottlenecks.

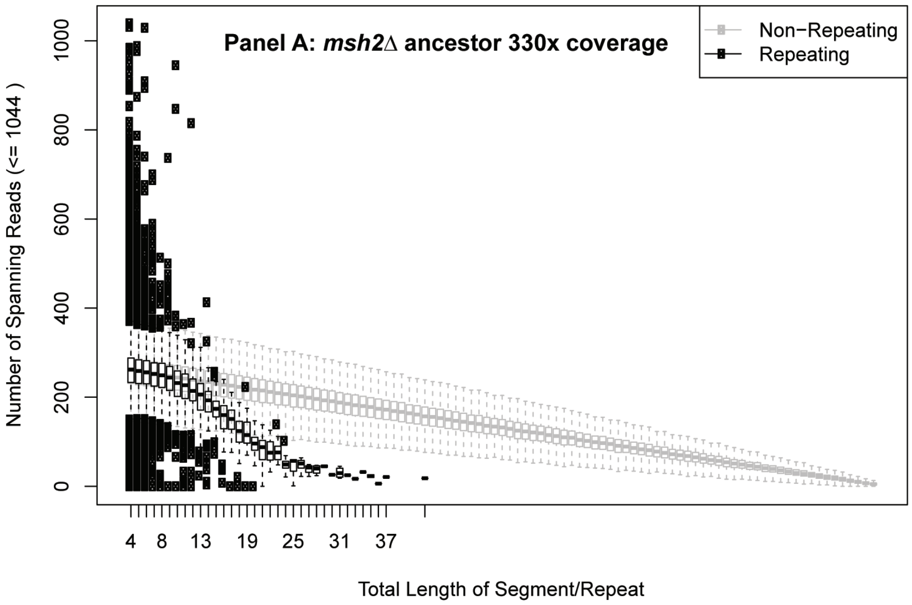

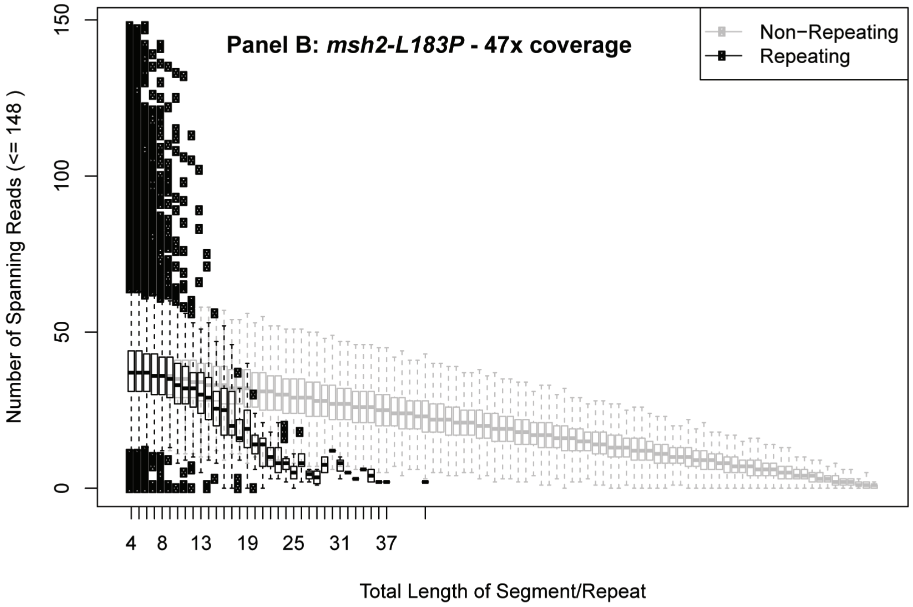

Figure S2 Spanning Read Counts for Repeats

Bedtools intersectBed (see Materials and Methods) was used to find the number of reads that overlap a microsatellite (repeating) region as well as non-repeating regions of varying length. Regions from chromosome XII (rDNA repeats) as well as regions with a read count >= 4x median were removed before plotting.

Table S1 Plasmids used in the study

Strain*	Plasmid Name	Relevant Genotype	Published Source
	pRS413	*HIS3 CEN/ARS*	(Sikorski and Hieter 1989)
AG17	pMSH2	*MSH2 HIS3 CEN/ARS*	(Gammie *et al.* 2007)
AG35	pMSH2-G688D	*msh2-G688D HIS3 CEN/ARS*	(Gammie *et al.* 2007)
AG86	pMSH2-D524Y	*msh2-D524Y HIS3 CEN/ARS*	(Gammie *et al.* 2007)
AG403	pMSH2-G770R	*msh2-G770R HIS3 CEN/ARS*	(Gammie *et al.* 2007)
AG421	pMSH2-A618V	*msh2-A618V HIS3 CEN/ARS*	(Gammie *et al.* 2007)
AG424	pMSH2-S742F	*msh2-S742F HIS3 CEN/ARS*	(Gammie *et al.* 2007)
AG486	pMSH2-L183P	*msh2-L183P HIS3 CEN/ARS*	(Arlow *et al.* 2013)
AG487	pMSH2-P640T	*msh2-P640T HIS3 CEN/ARS*	(Arlow *et al.* 2013)
AG488	pMSH2-P689L	*msh2-P689L HIS3 CEN/ARS*	This study
AG495	pMSH2-C195Y	*msh2-C195Y HIS3 CEN/ARS*	(Arlow *et al.* 2013)
AG496	pMSH2-R542L	*msh2-R542L HIS3 CEN/ARS*	(Arlow *et al.* 2013)
AG497	pMSH2-D621G	*msh2-D621G HIS3 CEN/ARS*	(Arlow *et al.* 2013)
AG499	pMSH2-S695P	*msh2-S695P HIS3 CEN/ARS*	This study
AG507	pMSH2-C345F	*msh2-C345F HIS3 CEN/ARS*	(Arlow *et al.* 2013)
AG545	pMSH2-R657G	*msh2-R657G HIS3 CEN/ARS*	This study
AG546	pMSH2-G693R	*msh2-G693R HIS3 CEN/ARS*	This study
AG547	pMSH2-T743K	*msh2-T743K HIS3 CEN/ARS*	This study

***All plasmids from the Gammie laboratory (Princeton University) except for pRS413

Table S2 Sequencing Coverage

Relevant Genotype	Description	Generation	Coverage
MSH2	genomic WT ancestor	0	299 x
MSH2	genomic WT passaged	~170	194 x
*msh2*Δ	Null ancestor	0	330 x
*msh2*Δ + pMSH2	CEN WT passaged	~170	165 x
*msh2*Δ + pRS413	Null passaged	~170	76 x
*msh2*Δ + pMSH2-L183P	Allele passaged	~170	47 x
*msh2*Δ + pMSH2-C195Y	Allele passaged	~170	147 x
*msh2*Δ + pMSH2-C345F	Allele passaged	~170	133 x
*msh2*Δ + pMSH2-D524Y	Allele passaged	~170	220 x
*msh2*Δ + pMSH2-R542L	Allele passaged	~170	145 x
*msh2*Δ + pMSH2-A618V	Allele passaged	~170	94 x
*msh2*Δ + pMSH2-D621G	Allele passaged	~170	255 x
*msh2*Δ + pMSH2-P640T	Allele passaged	~170	103 x
*msh2*Δ + pMSH2-R657G	Allele passaged	~170	118 x
*msh2*Δ + pMSH2-G688D	Allele passaged	~170	166 x
*msh2*Δ + pMSH2-P689L	Allele passaged	~170	188 x
*msh2*Δ + pMSH2-G693R	Allele passaged	~170	60 x
*msh2*Δ + pMSH2-S742F	Allele passaged	~170	153 x
*msh2*Δ + pMSH2-T743K	Allele passaged	~170	192 x
*msh2*Δ + pMSH2-S695P	Allele passaged	~170	99 x
*msh2*Δ + pMSH2-G770R	Allele passaged	~170	175 x

Table S3 Parameters for Mapping with BWA for Illumina

Input Parameter	Value
Conditional (genomeSource)	0
Select a reference genome	w303_draft
Conditional (paired)	0
Conditional (params)	1
Maximum edit distance	0
Fraction of missing alignments given 2% uniform base error rate	0.04
Maximum number of gap opens	1
Maximum number of gap extensions	-1
Disallow long deletion within [value] bp towards the 3'-end	16
Disallow insertion/deletion within [value] bp towards the end	0
Number of first subsequences to take as seed	-1
Maximum edit distance in the seed	2
Mismatch penalty	3
Gap open penalty	5
Gap extension penalty	4
Proceed with suboptimal alignments even if the top hit is a repeat	False
Disable iterative search	False
Maximum number of alignments to output in the XA tag for reads paired properly	3
Maximum number of alignments to output in the XA tag for disconcordant read pairs (excluding singletons)	10
Maximum insert size for a read pair to be considered as being mapped properly	500
Maximum occurrences of a read for pairing	100000
Quality threshold for read trimming down to 35bp	0

Table S4   Freebayes Parameters

Input Parameter	Value
Bam Alignment File	passaged
Additional Bam Alignment File	ancestor
Select Reference Genome	w303_draft
Freebayes Settings to Use	full
Theta	0.001
Ploidy	1
Pooled	False
Probability of variant threshold	0.0001
Show Reference Repeats	False
Ignore SNP alleles	False
Ignore insertion and deletion alleles	False
Ignore multi-nuceotide polymorphisms, MNPs	False
Ignore complex events (composites of other classes)	False
Use Best N Alleles	0
Left align indels	True
Base alignment quality (BAQ) adjustment	True
Use Reference Allele	False
Reference Ploidy	Haploid
Assign mapping quality of Q to the reference allele at each site	100
Reference Base Quality	60
Use duplicate reads	False
Minimum Mapping Quality	30
Minimum Base Quality	20
No Filters	True
Indel Exclusion Window	-1
Minimum Alternative Fraction	0.0
Minimum Alternative Count	1
Minimum Alternative Total	1
Minimum Coverage	0
Posterior Integration Limit N	1
Posterior Integration Limit M	3

Table S5 Unique mutations in the *msh2*Δ ancestor

Chr	Position	Original	Change	Gene	Consequence	Description of gene from SGD (Cherry *et al.* 1997)
chr03	196894	C	A	*PHO87*	missense D>Y	Low-affinity inorganic phosphate (Pi) transporter; involved in activation of PHO pathway; expression is independent of Pi concentration and Pho4p activity; contains 12 membrane-spanning segments; *PHO87* has a paralog, *PHO90*, that arose from the whole genome duplication
chr04	32896	C	G	*PHO13*	missense Q>E	Alkaline phosphatase specific for p-nitrophenyl phosphate; also has protein phosphatase activity
chr04	673259	T	TA	*TRS85*	frameshift	Subunit of TRAPPIII (transport protein particle), a multimeric guanine nucleotide-exchange factor for Ypt1p, required for membrane expansion during autophagy and the CVT pathway; directs Ypt1p to the PAS; late post-replication meiotic role
chr05	349345	AT	A	intergenic		
chr07	106429	G	T	*CHC1*	missense T>K	Clathrin heavy chain, subunit of the major coat protein involved in intracellular protein transport and endocytosis; two heavy chains form the clathrin triskelion structural component; the light chain (*CLC1*) is thought to regulate function
chr07	395930	TA	T	intergenic		
chr07	609723	GA	G	intergenic		
chr07	798380	AT	A	intergenic		
chr10	164291	AT	A	*AIM23*	frameshift, lose 4 amino acids	Mitochondrial translation initiation factor 3 (IF3, mIF3); evolutionarily conserved; binds to E. coli ribosomes in vitro; null mutant displays severe respiratory growth defect and elevated frequency of mitochondrial genome loss
chr12	340,658	C	T	*ICT1*	missense E>K	Lysophosphatidic acid acyltransferase; responsible for enhanced phospholipid synthesis during organic solvent stress; null displays increased sensitivity to Calcofluor white; highly expressed during organic solvent stress; *ICT1* has a paralog, *ECM18*, that arose from the whole genome duplication
chr15	92494	TA	T	*RPS19A*	frameshift within intron	Protein component of the small (40S) ribosomal subunit; required for assembly and maturation of pre-40 S particles; homologous to mammalian ribosomal protein S19, no bacterial homolog; mutations in human RPS19 are associated with Diamond Blackfan anemia; *RPS19A* has a paralog, *RPS19B*, that arose from the whole genome duplication
chr15	123982	CT	C	intergenic		
chr15	137881	TA	T	intergenic		
chr15	659986	G	T	*ALE1*	nonsense Y>stop	Broad-specificity lysophospholipid acyltransferase, part of MBOAT family of membrane-bound O-acyltransferases; key component of Lands cycle; may have role in fatty acid exchange at sn-2 position of mature glycerophospholipids
chr15	825192	G	A	intergenic		

Table S6 Mutation Spectra of Missense Variants

Functional Domain	Relevant Genotype	Single Base Pair Substitutions	Insertions or Deletions Di-or Tri Nucleotides	Insertions or Deletions Homopolymers	*p* value compared to null
Null	*msh2Δ*	7	6	134	0.47
Structural Integrity	*msh2-A618V*	8	11	98	0.02
	msh2-R657G	6	7	135	0.29
	msh2-L183P	7	12	131	0.03
	*msh2-C195Y**	15	7	151	0.81
	msh2-C345F	16	12	168	0.34
	*msh2-D621G**	12	5	139	0.83
	msh2-P640T	10	8	117	0.59
DNA binding	*msh2-R542L*	4	3	132	0.05
	msh2-D524Y	14	13	137	0.04
ATPase	*msh2-G688D*	15	12	127	0.04
	msh2-G693R	9	12	134	0.07
	*msh2-S695P**	14	9	150	0.73
	msh2-S742F	9	13	143	0.04
	msh2-T743K	5	9	137	0.10
	msh2-G770R	7	7	140	0.40

*plasmid rearrangement, data combined with *msh2Δ* data for the null control in Fisher Exact tests

References for Supplementary Material

Arlow, T., K. Scott, A. Wagenseller and A. Gammie, 2013 Proteasome inhibition rescues clinically significant unstable variants of the mismatch repair protein Msh2. Proc Natl Acad Sci U S A **110:** 246-251.

Cherry, J. M., C. Ball, S. Weng, G. Juvik, R. Schmidt *et al.*, 1997 Genetic and physical maps of *Saccharomyces cerevisiae*. Nature **387:** 67-73.

Gammie, A. E., N. Erdeniz, J. Beaver, B. Devlin, A. Nanji *et al.*, 2007 Functional characterization of pathogenic human MSH2 missense mutations in Saccharomyces cerevisiae. Genetics **177:** 707-721.

Sikorski, R. S., and P. Hieter, 1989 A system of shuttle vectors and yeast host strains designed for efficient manipulation of DNA in *Saccharomyces cerevisiae*. Genetics **122:** 19-27.
