# Supplementary material for: Mutation Rates, Spectra, and Genome-Wide Distribution of Spontaneous Mutations in Mismatch Repair Deficient Yeast
Source: G3 (Bethesda). 2013 Sep 1;3(9):1453–65. doi: 10.1534/g3.113.006429 (PMC3755907; doi:10.1534/g3.113.006429)
Supplement: Supporting Information [file supp_g3.113.006429_FigureS1.pdf]

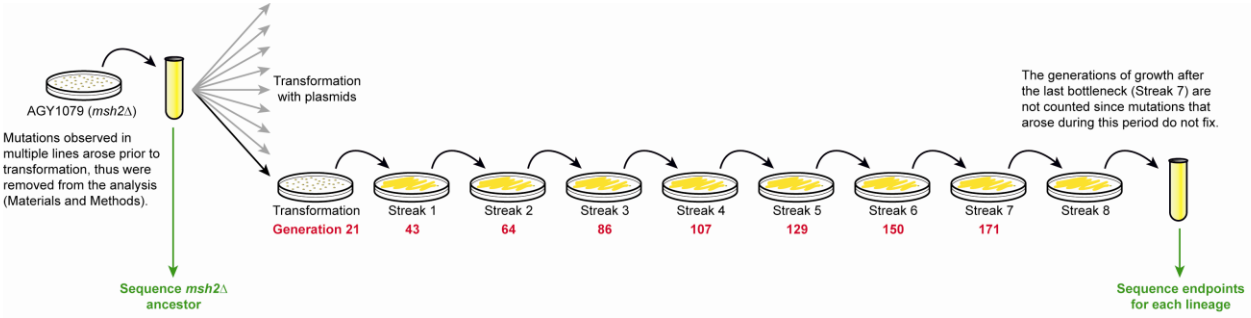

Figure S1: Schematic of Experimental Design. A single colony of AGY1079 was picked and transformed with *msh2*-containing plasmids. A single colony from each transformation was selected to begin the mutation accumulation experiment. Strains were passaged by streaking for single colonies every two days. We estimate ~21 generations of growth between bottlenecks.
