# Supplementary material for: Mutation Rates, Spectra, and Genome-Wide Distribution of Spontaneous Mutations in Mismatch Repair Deficient Yeast
Source: G3 (Bethesda). 2013 Sep 1;3(9):1453–65. doi: 10.1534/g3.113.006429 (PMC3755907; doi:10.1534/g3.113.006429)
Supplement: Supporting Information [file supp_g3.113.006429_FigureS2.pdf]

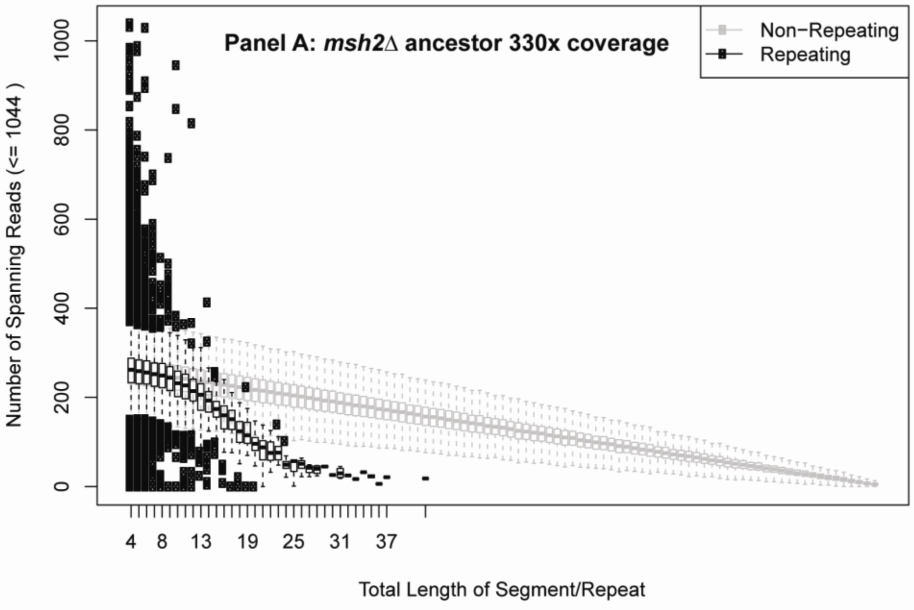

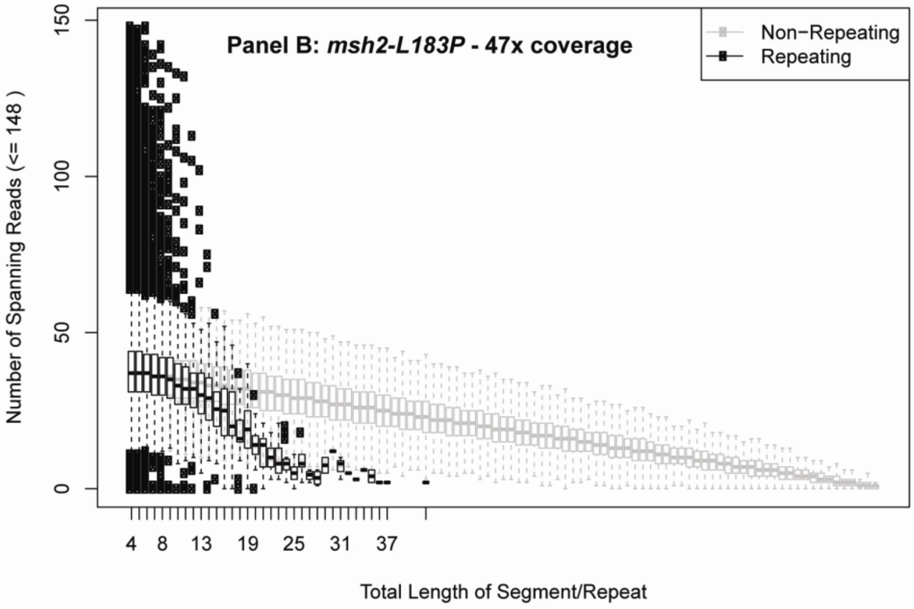

Figure S2 Spanning Read Counts for Repeats

Bedtools intersectBed (see Materials and Methods) was used to find the number of reads that overlap a microsatellite (repeating) region as well as non-repeating regions of varying length. Regions from chromosome XII (rDNA repeats) as well as regions with a read count >= 4x median were removed before plotting.
