# Supplementary material for: Mutation Rates, Spectra, and Genome-Wide Distribution of Spontaneous Mutations in Mismatch Repair Deficient Yeast
Source: G3 (Bethesda). 2013 Sep 1;3(9):1453–65. doi: 10.1534/g3.113.006429 (PMC3755907; doi:10.1534/g3.113.006429)
Supplement: Supporting Information [file supp_g3.113.006429_TableS1.pdf]

Table S1 Plasmids used in the study

Strain*	Plasmid Name	Relevant Genotype	Published Source
	pRS413	*HIS3 CEN/ARS*	(Sikorski and Hieter 1989)
AG17	pMSH2	*MSH2 HIS3 CEN/ARS*	(Gammie *et al.* 2007)
AG35	pMSH2-G688D	*msh2-G688D HIS3 CEN/ARS*	(Gammie *et al.* 2007)
AG86	pMSH2-D524Y	*msh2-D524Y HIS3 CEN/ARS*	(Gammie *et al.* 2007)
AG403	pMSH2-G770R	*msh2-G770R HIS3 CEN/ARS*	(Gammie *et al.* 2007)
AG421	pMSH2-A618V	*msh2-A618V HIS3 CEN/ARS*	(Gammie *et al.* 2007)
AG424	pMSH2-S742F	*msh2-S742F HIS3 CEN/ARS*	(Gammie *et al.* 2007)
AG486	pMSH2-L183P	*msh2-L183P HIS3 CEN/ARS*	(Arlow *et al.* 2013)
AG487	pMSH2-P640T	*msh2-P640T HIS3 CEN/ARS*	(Arlow *et al.* 2013)
AG488	pMSH2-P689L	*msh2-P689L HIS3 CEN/ARS*	This study
AG495	pMSH2-C195Y	*msh2-C195Y HIS3 CEN/ARS*	(Arlow *et al.* 2013)
AG496	pMSH2-R542L	*msh2-R542L HIS3 CEN/ARS*	(Arlow *et al.* 2013)
AG497	pMSH2-D621G	*msh2-D621G HIS3 CEN/ARS*	(Arlow *et al.* 2013)
AG499	pMSH2-S695P	*msh2-S695P HIS3 CEN/ARS*	This study
AG507	pMSH2-C345F	*msh2-C345F HIS3 CEN/ARS*	(Arlow *et al.* 2013)
AG545	pMSH2-R657G	*msh2-R657G HIS3 CEN/ARS*	This study
AG546	pMSH2-G693R	*msh2-G693R HIS3 CEN/ARS*	This study
AG547	pMSH2-T743K	*msh2-T743K HIS3 CEN/ARS*	This study

*****All plasmids from the Gammie laboratory (Princeton University) except for pRS413
