# Supplementary material for: Mutation Rates, Spectra, and Genome-Wide Distribution of Spontaneous Mutations in Mismatch Repair Deficient Yeast
Source: G3 (Bethesda). 2013 Sep 1;3(9):1453–65. doi: 10.1534/g3.113.006429 (PMC3755907; doi:10.1534/g3.113.006429)
Supplement: Supporting Information [file supp_g3.113.006429_TableS2.pdf]

Table S2 Sequencing Coverage

Relevant Genotype	Description	Generation	Coverage
MSH2	genomic WT ancestor	0	299 x
MSH2	genomic WT passaged	~170	194 x
*msh2*Δ	Null ancestor	0	330 x
*msh2*Δ + pMSH2	CEN WT passaged	~170	165 x
*msh2*Δ + pRS413	Null passaged	~170	76 x
*msh2*Δ + pMSH2-L183P	Allele passaged	~170	47 x
*msh2*Δ + pMSH2-C195Y	Allele passaged	~170	147 x
*msh2*Δ + pMSH2-C345F	Allele passaged	~170	133 x
*msh2*Δ + pMSH2-D524Y	Allele passaged	~170	220 x
*msh2*Δ + pMSH2-R542L	Allele passaged	~170	145 x
*msh2*Δ + pMSH2-A618V	Allele passaged	~170	94 x
*msh2*Δ + pMSH2-D621G	Allele passaged	~170	255 x
*msh2*Δ + pMSH2-P640T	Allele passaged	~170	103 x
*msh2*Δ + pMSH2-R657G	Allele passaged	~170	118 x
*msh2*Δ + pMSH2-G688D	Allele passaged	~170	166 x
*msh2*Δ + pMSH2-P689L	Allele passaged	~170	188 x
*msh2*Δ + pMSH2-G693R	Allele passaged	~170	60 x
*msh2*Δ + pMSH2-S742F	Allele passaged	~170	153 x
*msh2*Δ + pMSH2-T743K	Allele passaged	~170	192 x
*msh2*Δ + pMSH2-S695P	Allele passaged	~170	99 x
*msh2*Δ + pMSH2-G770R	Allele passaged	~170	175 x
