# Supplementary material for: Mutation Rates, Spectra, and Genome-Wide Distribution of Spontaneous Mutations in Mismatch Repair Deficient Yeast
Source: G3 (Bethesda). 2013 Sep 1;3(9):1453–65. doi: 10.1534/g3.113.006429 (PMC3755907; doi:10.1534/g3.113.006429)
Supplement: Supporting Information [file supp_g3.113.006429_TableS3.pdf]

Table S3   Parameters for Mapping with BWA for Illumina

Input Parameter	Value
Conditional (genomeSource)	0
Select a reference genome	w303_draft
Conditional (paired)	0
Conditional (params)	1
Maximum edit distance	0
Fraction of missing alignments given 2% uniform base error rate	0.04
Maximum number of gap opens	1
Maximum number of gap extensions	-1
Disallow long deletion within [value] bp towards the 3'-end	16
Disallow insertion/deletion within [value] bp towards the end	0
Number of first subsequences to take as seed	-1
Maximum edit distance in the seed	2
Mismatch penalty	3
Gap open penalty	5
Gap extension penalty	4
Proceed with suboptimal alignments even if the top hit is a repeat	False
Disable iterative search	False
Maximum number of alignments to output in the XA tag for reads paired properly	3
Maximum number of alignments to output in the XA tag for disconcordant read pairs (excluding singletons)	10
Maximum insert size for a read pair to be considered as being mapped properly	500
Maximum occurrences of a read for pairing	100000
Quality threshold for read trimming down to 35bp	0
