# Supplementary material for: Mutation Rates, Spectra, and Genome-Wide Distribution of Spontaneous Mutations in Mismatch Repair Deficient Yeast
Source: G3 (Bethesda). 2013 Sep 1;3(9):1453–65. doi: 10.1534/g3.113.006429 (PMC3755907; doi:10.1534/g3.113.006429)
Supplement: Supporting Information [file supp_g3.113.006429_TableS4.pdf]

Table S4 Freebayes Parameters

Input Parameter	Value
Bam Alignment File	passaged
Additional Bam Alignment File	ancestor
Select Reference Genome	w303_draft
Freebayes Settings to Use	full
Theta	0.001
Ploidy	1
Pooled	False
Probability of variant threshold	0.0001
Show Reference Repeats	False
Ignore SNP alleles	False
Ignore insertion and deletion alleles	False
Ignore multi-nuceotide polymorphisms, MNPs	False
Ignore complex events (composites of other classes)	False
Use Best N Alleles	0
Left align indels	True
Base alignment quality (BAQ) adjustment	True
Use Reference Allele	False
Reference Ploidy	Haploid
Assign mapping quality of Q to the reference allele at each site	100
Reference Base Quality	60
Use duplicate reads	False
Minimum Mapping Quality	30
Minimum Base Quality	20
No Filters	True
Indel Exclusion Window	-1
Minimum Alternative Fraction	0.0
Minimum Alternative Count	1
Minimum Alternative Total	1
Minimum Coverage	0
Posterior Integration Limit N	1
Posterior Integration Limit M	3
