# Supplementary material for: Mutation Rates, Spectra, and Genome-Wide Distribution of Spontaneous Mutations in Mismatch Repair Deficient Yeast
Source: G3 (Bethesda). 2013 Sep 1;3(9):1453–65. doi: 10.1534/g3.113.006429 (PMC3755907; doi:10.1534/g3.113.006429)
Supplement: Supporting Information [file supp_g3.113.006429_TableS5.pdf]

Table S5 Unique mutations in the *msh2*Δ ancestor

Chr	Position	Original	Change	Gene	Consequence	Description of gene from SGD (Cherry *et al.* 1997)
chr03	196894	C	A	*PHO87*	missense D>Y	Low-affinity inorganic phosphate (Pi) transporter; involved in activation of PHO pathway; expression is independent of Pi concentration and Pho4p activity; contains 12 membrane-spanning segments; *PHO87* has a paralog, *PHO90*, that arose from the whole genome duplication
chr04	32896	C	G	*PHO13*	missense Q>E	Alkaline phosphatase specific for p-nitrophenyl phosphate; also has protein phosphatase activity
chr04	673259	T	TA	*TRS85*	frameshift	Subunit of TRAPPIII (transport protein particle), a multimeric guanine nucleotide-exchange factor for Ypt1p, required for membrane expansion during autophagy and the CVT pathway; directs Ypt1p to the PAS; late post-replication meiotic role
chr05	349345	AT	A	intergenic		
chr07	106429	G	T	*CHC1*	missense T>K	Clathrin heavy chain, subunit of the major coat protein involved in intracellular protein transport and endocytosis; two heavy chains form the clathrin triskelion structural component; the light chain (*CLC1*) is thought to regulate function
chr07	395930	TA	T	intergenic		
chr07	609723	GA	G	intergenic		
chr07	798380	AT	A	intergenic		
chr10	164291	AT	A	*AIM23*	frameshift, lose 4 amino acids	Mitochondrial translation initiation factor 3 (IF3, mIF3); evolutionarily conserved; binds to E. coli ribosomes in vitro; null mutant displays severe respiratory growth defect and elevated frequency of mitochondrial genome loss
chr12	340,658	C	T	*ICT1*	missense E>K	Lysophosphatidic acid acyltransferase; responsible for enhanced phospholipid synthesis during organic solvent stress; null displays increased sensitivity to Calcofluor white; highly expressed during organic solvent stress; *ICT1* has a paralog, *ECM18*, that arose from the whole genome duplication
chr15	92494	TA	T	*RPS19A*	frameshift within intron	Protein component of the small (40S) ribosomal subunit; required for assembly and maturation of pre-40 S particles; homologous to mammalian ribosomal protein S19, no bacterial homolog; mutations in human RPS19 are associated with Diamond Blackfan anemia; *RPS19A* has a paralog, *RPS19B*, that arose from the whole genome duplication
chr15	123982	CT	C	intergenic		
chr15	137881	TA	T	intergenic		
chr15	659986	G	T	*ALE1*	nonsense Y>stop	Broad-specificity lysophospholipid acyltransferase, part of MBOAT family of membrane-bound O-acyltransferases; key component of Lands cycle; may have role in fatty acid exchange at sn-2 position of mature glycerophospholipids
chr15	825192	G	A	intergenic		
