# Supplementary material for: Mutation Rates, Spectra, and Genome-Wide Distribution of Spontaneous Mutations in Mismatch Repair Deficient Yeast
Source: G3 (Bethesda). 2013 Sep 1;3(9):1453–65. doi: 10.1534/g3.113.006429 (PMC3755907; doi:10.1534/g3.113.006429)
Supplement: Supporting Information [file supp_g3.113.006429_TableS6.pdf]

Table S6 Mutation Spectra of Missense Variants

Functional Domain	Relevant Genotype	Single Base Pair Substitutions	Insertions or Deletions Di-or Tri Nucleotides	Insertions or Deletions Homopolymers	*p* value compared to null
Null	*msh2*Δ	7	6	134	0.47
Structural Integrity	*msh2-A618V*	8	11	98	0.02
	msh2-R657G	6	7	135	0.29
	msh2-L183P	7	12	131	0.03
	*msh2-C195Y**	15	7	151	0.81
	msh2-C345F	16	12	168	0.34
	*msh2-D621G**	12	5	139	0.83
	msh2-P640T	10	8	117	0.59
DNA binding	*msh2-R542L*	4	3	132	0.05
	msh2-D524Y	14	13	137	0.04
ATPase	*msh2-G688D*	15	12	127	0.04
	msh2-G693R	9	12	134	0.07
	*msh2-S695P**	14	9	150	0.73
	msh2-S742F	9	13	143	0.04
	msh2-T743K	5	9	137	0.10
	msh2-G770R	7	7	140	0.40

*plasmid rearrangement, data combined with *msh2*Δ data for the null control in Fisher Exact tests
