# Supplementary material for: Mutation Rates, Spectra, and Genome-Wide Distribution of Spontaneous Mutations in Mismatch Repair Deficient Yeast
Source: G3 (Bethesda). 2013 Sep 1;3(9):1453–65. doi: 10.1534/g3.113.006429 (PMC3755907; doi:10.1534/g3.113.006429)
Supplement: Supporting Information [file supp_g3.113.006429_SupportingReferences.pdf]

References for Supplementary Material

Arlow, T., K. Scott, A. Wagenseller and A. Gammie, 2013 Proteasome inhibition rescues clinically significant unstable variants of the mismatch repair protein Msh2. Proc Natl Acad Sci U S A **110:** 246-251.

Cherry, J. M., C. Ball, S. Weng, G. Juvik, R. Schmidt *et al.*, 1997 Genetic and physical maps of *Saccharomyces cerevisiae*. Nature **387:** 67-73.

Gammie, A. E., N. Erdeniz, J. Beaver, B. Devlin, A. Nanji *et al.*, 2007 Functional characterization of pathogenic human MSH2 missense mutations in Saccharomyces cerevisiae. Genetics **177:** 707-721.

Sikorski, R. S., and P. Hieter, 1989 A system of shuttle vectors and yeast host strains designed for efficient manipulation of DNA in *Saccharomyces cerevisiae*. Genetics **122:** 19-27.
